# Years of life lost due to insufficient sleep and associated economic burden in China from 2010–18

**DOI:** 10.7189/jogh.14.04076

**Published:** 2024-04-05

**Authors:** Xumeng Yan, Fang Han, Haowei Wang, Zhihui Li, Ichiro Kawachi, Xiaoyu Li

**Affiliations:** 1 Department of Sociology, Tsinghua University, Beijing, China; 2 Department of Community Health Sciences, University of California, Los Angeles, California, USA; 3 Division of Sleep Medicine, Beijing University People’s Hospital, Beijing, China; 4 Department of Sociology and Aging Studies Institute, Maxwell School of Citizenship and Public Affairs, Syracuse University, Syracuse, New York, USA; 5 Vanke School of Public Health, Tsinghua University, Beijing, China; 6 Department of Social and Behavioural Sciences, Harvard T.H. Chan School of Public Health, Boston, Massachusetts, USA

## Abstract

**Background:**

Research on the health and economic costs due to insufficient sleep remains scant in developing countries. In this study we aimed to estimate the years of life lost (YLLs) due to short sleep and quantify its economic burden in China.

**Methods:**

We estimated both individual and aggregate YLLs due to short sleep (ie, ≤6 hours) among Chinese adults aged 20 years or older by sex and five-year age groups in 2010, 2014, and 2018. YLL estimates were derived from 1) the prevalence of short sleep using three survey waves of the China Family Panel Studies, 2) relative mortality risks from meta-analyses, and 3) life tables in China. YLL was the difference between the estimated life expectancy of an individual in the short sleep category vs in the recommended sleep category. We estimated the economic cost using the human capital approach.

**Results:**

The sample sizes of the three survey waves were 31 393, 31 207, and 28 618. Younger age groups and men had more YLLs due to short sleep compared to their counterparts. For individuals aged 20–24, men had an average YLL of nearly 0.95, in contrast to the approximate 0.75 in women across the observed years of 2010, 2014, and 2018. The trend in individual YLLs remained consistent over these years. In aggregate, China experienced a rise from 66.75 million YLLs in 2010 to 95.29 million YLLs in 2014, and to 115.05 million YLLs in 2018. Compared to 2010 (USD 191.83 billion), the associated economic cost in 2014 increased to USD 422.24 billion, and the cost in 2018 more than tripled (USD 628.15 billion). The percentage of cost to Chinese gross domestic product in corresponding years was 3.23, 4.09, and 4.62%.

**Conclusions:**

Insufficient sleep is associated with substantial YLLs in China, potentially impacting the population’s overall life expectancy. The escalating economic toll attributed to short sleep underscores the urgent need for public health interventions to improve sleep health at the population level.

Insufficient sleep poses a burden to public health due to its multiple negative outcomes. Previous studies have shown that short sleep duration increases the risk of all-cause mortality [1], as well as the risks of many chronic conditions such as coronary heart disease, stroke, diabetes mellitus, hypertension, and obesity [2,3]. Lack of sleep not only leads to adverse health outcomes but also results in substantial economic consequences. The estimated annual cost due to insufficient sleep amounted to USD 411 billion in the USA in 2015–16 [4]. The estimated economic cost of inadequate sleep in Australia was USD 45.21 billion in 2016–17, representing 4.6% of all costs associated with the burden of disease [5].

As a rapidly developing economy, China has the largest population at risk of insufficient sleep. Chinese people are getting less sleep compared with a decade ago [6]. Although considerable research points to the importance of adequate sleep for health, factors such as the use of digital devices, long work hours, and sleep disorders often prevent people from meeting sleep recommendations [7]. According to the Chinese Sleep Research Report 2022, more than 60% of Chinese people sleep less than eight hours per day [6].

Data on the health and economic costs of insufficient sleep in China are scarce yet urgently needed to inform health services and public policies. The years of life lost (YLLs) due to premature mortality is a common way to measure the burden of a certain disease [8]. This measurement offers a more comprehensive assessment of the disproportionate mortality burden related to sleep duration, emphasising the economic impact of premature deaths, particularly at younger ages [7]. While the Global Burden of Disease Study publishes annual estimates of health loss from various diseases, injuries, and risk factors [9], inadequate sleep has not been listed yet.

Therefore, in this study, we aimed to estimate the temporal trends of YLLs due to short sleep and quantify their economic cost in China from 2010–18. We hope to translate the otherwise imperceptible health risks into economic indicators that are more intuitive and easy to understand for individuals and policymakers.

## METHODS

### Overview of the YLL estimation method

YLLs due to inadequate sleep were estimated using three sources of information: the estimated prevalence of sleep duration categories by sex and age groups, obtained from three waves of national survey data of Chinese adults; the relative risks of all-cause mortality within these sleep duration categories, obtained from meta-analyses; and the probability of death for men and women in each age group across adult life, obtained from China life tables published by the World Health Organization (WHO). The estimation approach is based on the methods developed by Fontaine and colleagues [10] and has been applied in various research estimating YLLs due to different health risk factors, including obesity, physical inactivity, and suboptimal sleep duration [11–13]. The analyses were conducted in *R*, version 4.3.1. (R Core Team, Vienna, Austria) and Microsoft Excel, version 16.77.1 (Microsoft Inc, Seattle, Washington, USA).

### Prevalence of sleep duration

We used the China Family Panel Studies data to estimate the prevalence of sleep duration categories among Chinese adults. The China Family Panel Studies, launched in 2010 and conducted every two years, is a nearly nationally representative longitudinal survey designed to collect high-quality information on a wide array of social phenomena in contemporary China [14]. In its 2010 baseline survey, it applied a multi-stage probability sampling technique to select 16 000 households across 25 provinces or administrative equivalents that represented 95% of the Chinese population [15]. Each selected household was required to fill out two family-level questionnaires on the family structure and relationships as well as basic information about the whole family. Furthermore, each member of the household was surveyed at the individual level, with individuals under 16 answering child questionnaires and those older than 16 answering adult questionnaires [16]. This study focused on the individual-level data from adults during the 2010, 2014, and 2018 survey waves for sleep duration prevalence estimation. In 2010, the adult questionnaire asked about the average hours spent sleeping per day on weekdays and weekends. Starting in 2014, the adult questionnaire collected information on self-reported sleep duration through two sets of questions: ‘Normally, how many hours do you sleep per day on weekdays/weekends?’ for individuals who were either employed or in school, and ‘Normally, how many hours do you sleep per day?’ for individuals who were neither employed nor in school. For individuals who reported sleep duration on weekdays and weekends, we used a weekday/weekend (five/two days) weighted approach to calculate their average sleep duration per day; otherwise, we used their reported value as the average sleep duration per day. Observations with values <3 hours or >13 hours were excluded following prior sleep studies’ approaches [17]. In consistency with the life table statistics, we restricted the analytic samples to adults aged 20 and older. The sample sizes were 31 393 (2010), 31 207 (2014), and 28 618 (2018).

The definitions of short and long sleep duration are inconsistent in previous studies. According to the United States National Sleep Foundation, adults aged 18–64 are recommended to get seven to nine hours of sleep, and adults aged 65 or older are recommended to get seven to eight hours of sleep [18,19]. In meta-analyses, the most widely used definitions are ≤6 hours for short sleep and ≥9 hours for long sleep, with the most common reference group being seven to eight hours [2,20,21]. This categorisation is particularly prevalent in research focusing on the Chinese population [22,23]. Aligning with these widely accepted categorisations for consistency in comparison, we defined short sleep duration as sleeping ≤6 hours per day and long sleep duration as ≥9 hours per day, thereby setting six to nine hours to delineate the normal sleep duration reference group, excluding its endpoints. For each sex and five-year age group, we estimated the proportion of individuals within each sleep duration category.

### Relative risks of all-cause mortality within sleep duration categories

The relative risks (RRs) of all-cause mortality were obtained from recently published meta-analyses. For adults aged 20–64 years, the pooled RR for short sleep duration was 1.12 (95% confidence interval (CI) = 1.06–1.18) [2] and the pooled RR for long sleep duration was 1.39 (95% CI = 1.31–1.47) [20]. For adults aged 65 years or older, the pooled RR for short sleep duration was 1.07 (95% CI = 1.03–1.11), and the pooled RR for long sleep duration was 1.34 (95% CI = 1.24–1.44) [20,21]. The RRs were used to calculate the adjustment factor of death probability. The adjustment factor equalled the RR of mortality associated with the given sleep duration category divided by the sum of the product of the sex- and age-group-specific proportion of people in each sleep duration category and their respective RRs of mortality [11].

### Individual YLLs

WHO publishes life tables by country every five years. Leveraging the available data sources, we used life tables in China published by WHO in 2010, 2015, and 2019 to approximate the death probabilities in 2010, 2014, and 2018. WHO life tables provided information on the probability of death by sex and five-year age groups starting at the age of 20 years [24]. For each five-year age interval, we obtained an estimate of the probability of death conditional on having lived to the start of that interval. Then, we multiplied the probability of death by the adjustment factor to obtain the sex- and age-group-specific death probability conditional on being in a specific sleep duration category [12,13]. Finally, we used the adjusted probability of death to calculate the adjusted life expectancy for each sleep duration category by sex and age group. YLLs due to short sleep were the difference in life expectancy between the short and appropriate sleep categories [13]. Because WHO reported death probabilities by age groups of 15–19 years, 20–24 years, etc., and the sleep duration recommendations were different for teens below 18 years and adults 18 years or older, we were unable to calculate the adjusted death probability for the group aged 15–19 years. Therefore, our age-group-based estimation was performed on individuals aged 20 or above.

### Aggregate YLLs

We used the China Statistical Yearbook published by the National Bureau of Statistics of China in 2011, 2015, and 2019 to obtain population estimates in 2010, 2014, and 2018 by sex and five-year age group [25]. The aggregate YLLs of the Chinese population were estimated by multiplying sex- and age-group-specific estimates of individual YLLs each year times the China population estimates in that year times the estimated prevalence of inadequate sleep by sex and age groups.

### The economic cost of aggregate YLLs due to short sleep

Under the assumption of human capital approach, the economic value of an individual is determined by labour earnings from productive activity [26]. According to the human capital approach, we adopted a common estimation strategy that multiplied the aggregate YLLs by per capita gross domestic product (GDP) in that year [25–28], incorporating productivity weights for different age groups to reflect differences in economic contribution [29]. Productivity weights for people aged 20–44, 45–59, and 60+ were defined as 0.75, 0.80, and 0.10 [29,30]. Additionally, we converted the monetary value into a percentage of the total GDP, offering a relative measure to facilitate a comparable understanding of the magnitude of the economic cost.

## RESULTS

**Figure 1:**
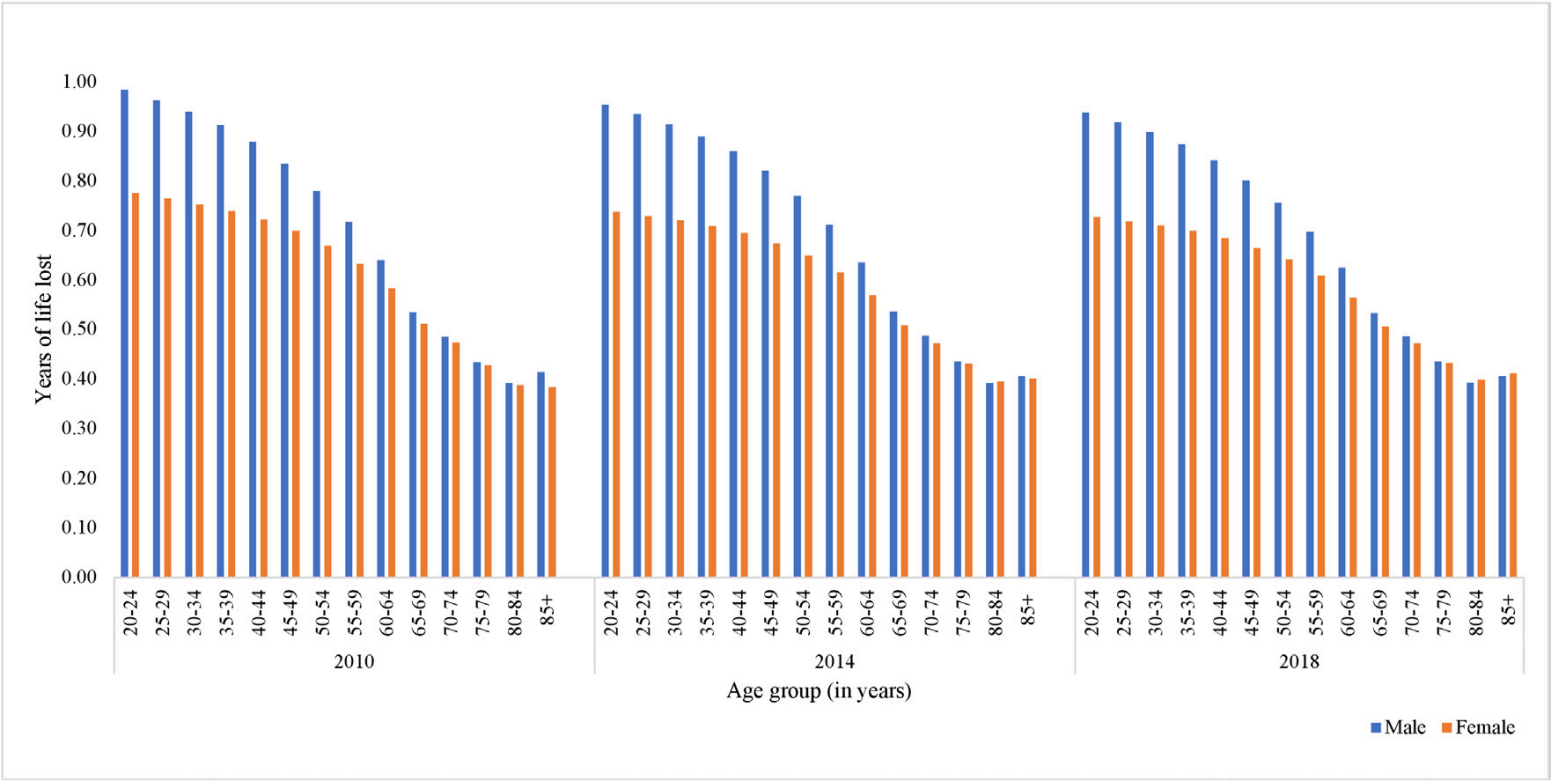
Individual years of life lost due to short sleep, by sex and age group from 2010–18.

On average, 9.7, 14.5, and 17.1% of Chinese adults have reported short sleep in 2010, 2014, and 2018. From 2010–18, the patterns of individual YLLs for Chinese adults with inadequate sleep duration (compared to individuals with appropriate sleep duration) were generally stable, with more YLLs observed at younger ages for both men and women and more YLLs for men than women in almost all age groups (**Figure 1**). The gap in YLLs between men and women narrowed at older ages. From 2010–18, individual YLLs, due to short sleep, slightly decreased for both sexes aged 20–64. For example, men aged 20–24 who had short sleep had an estimated life expectancy that was 0.98 years shorter than their counterparts with appropriate sleep duration in 2010 and 0.94 years shorter in 2018, whereas women aged 20–24 lost 0.78 years of life to short sleep in 2010 and 0.73 years in 2018. For men and women aged 85+ years, the YLLs remained largely unchanged across survey years, with both sexes losing 0.41 years of life due to short sleep in 2018 (Table S1 in the **Online Supplementary Document**).

**Figure 2:**
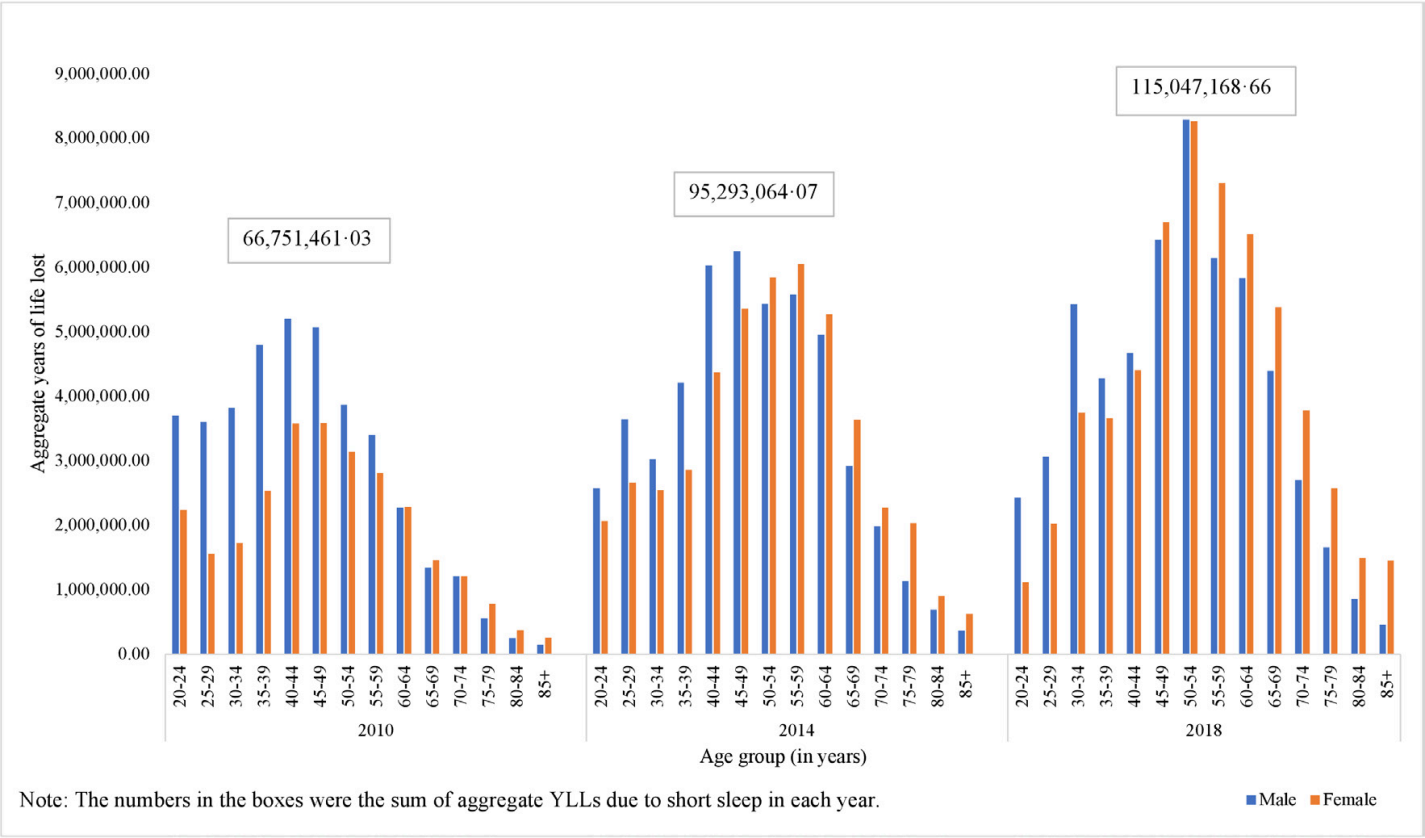
Aggregate years of life lost due to short sleep by sex and age group from 2010–18.

The results of aggregate YLLs showed that for the 2010 Chinese adult population, short sleep was associated with 66.8 million YLLs in total. The aggregate YLLs increased to 95.3 million in 2014 and 115.0 million in 2018 (**Figure 2**). In 2010 and 2014, men had more YLLs due to short sleep than women (39.2 million for men and 27.5 million for women in 2010; 48.8 million for men and 46.5 million for women in 2014). Yet in 2018, women lost more years of life than men (56.6 million for men and 58.4 million for women). At younger ages, men had more aggregate YLLs compared to women, while at older ages, women had more aggregate YLLs compared to men (Table S1 in the **Online Supplementary Document**).

Using Chinese GDP, per capita GDP, and age-group-adjusted productivity weights, we estimated the economic cost of aggregate YLLs for each year of the survey. In 2010, the estimated economic cost of total YLLs due to short sleep was USD 191.83 billion, which constituted 3.23% of the 2010 Chinese total GDP. In 2014, the estimated economic cost was USD 422.24 billion, representing 4.09% of the 2014 total GDP of China. The estimated economic cost associated with short sleep in 2018 rose to USD 628.15 billion, which was 4.62% of the 2018 Chinese total GDP [25,31].

## DISCUSSION

Our study is among the first to quantify the YLLs due to insufficient sleep and the associated economic burden in China. The analyses provided information on the temporal trends of the estimated individual and aggregate YLLs and translated it into the monetary value of the economic burden of short sleep on the country level.

Our results on estimated YLLs due to short sleep showed similar patterns with previous findings in other countries. Europeans aged 50–75 who slept inadequately were estimated to lose 1.1 years of healthy and disease-free life expectancy [32], whilst our study estimated 0.76 and 0.64 YLLs for Chinese men and women aged 50–54 in 2018. A recent study that used similar estimation approaches to ours reported 1.24 YLLs due to short sleep for Canadians at the age of 20 [13]. Our estimates were a bit lower, with 0.94 and 0.73 YLLs for Chinese men and women aged 20–24, respectively. The differences may arise because the study on Canadian adults reported estimated years of life gained for sleeping recommended hours. In other words, they estimated YLLs for short and long sleep duration. Our study, however, only included estimates of YLLs for short sleep duration, as the association between long sleep duration and premature mortality could be confounded by reversed causation. Consistent with the findings from the Canadian population, we observed that more YLLs were lost in younger groups and that men experienced more YLLs due to short sleep compared to women [13]. Particularly, younger adult males lost nearly a full year of potential life due to short sleep, an alarming figure when juxtaposed with the marginal annual increases in overall life expectancy for the Chinese population (ie, an annual increase of only 0.2–0.3 years over the last decade) [33].

Our estimate of economic cost attributable to short sleep in 2018, in terms of percentages, was similar in magnitude but slightly higher when compared to reported figures elsewhere. Compared to our estimate of 4.62% of GDP in 2018, insufficient sleep cost 3.08% of GDP in Japan in 2020, 2.40% in the USA in 2020, and 3.7% in Australia in 2016 [4,5]. Such discrepancy may be due to these studies’ varied economic cost estimation methods. Our study applied age-adjusted productivity weights to per capita GDP for cost estimation, while other studies used average earnings or macro-econometric modelling. The age distribution and demographics of the workforce can also affect how YLLs associated with short sleep translate into economic cost. Furthermore, sleep patterns are influenced by a complex interplay of cultural, environmental, and possibly genetic factors [34]. For example, evidence has suggested that in some East Asian cultures, reduced sleep is often viewed as necessary for achieving maximal performance, with less emphasis placed on the potential negative health consequences [35]. Even within broadly similar cultures, environmental factors such as air pollution, alongside lifestyle choices, may contribute to different manifestations of sleep problems [36]. Such cultural dimensions of sleep health highlight the uniqueness of the Chinese context and the necessity of considering the local context when assessing the public health implications of short sleep and its economic consequences.

Despite different estimation approaches and years, our results were comparable to the estimated economic cost of other health risk factors in China. For instance, the total cost of physical inactivity was estimated to be USD 6.7 billion in 2007 [37] and the total cost of smoking amounted to USD 28.9 billion in 2008 [38]. These studies yielded substantially lower estimates of economic cost, mainly because they both relied on population-attributable fractions to calculate the direct and indirect costs induced by certain chronic diseases related to physical inactivity or smoking. On the contrary, our study estimated the economic burden of potential YLLs for short sleepers by comparing their expected life expectancy with their counterparts who slept adequately.

From 2010–18, individual YLLs remained mostly stable, with slight decreases among younger populations. However, aggregate YLLs showed a substantial increase of nearly 2-fold, from 66.75 million to 115.05 million. This growth was largely attributable to the growing size of older populations, which have a higher prevalence of short sleep duration. The estimated economic burden of short sleep in China also underwent a sharp increase from USD 191.83 billion in 2010 to USD 628.15 billion in 2018, with the represented proportion of Chinese GDP changing from 3.23% to 4.62%. The higher prevalence of short sleepers, in addition to the larger population size, may explain the rise and the higher GDP per capita in 2018 compared to 2010. These results suggested that with a growing ageing population [39], the increasing prevalence of short sleep would continue to pose a challenge to China’s health care cost and economy. The growing trend highlights the need to develop targeted programs to help people sleep adequate hours, live longer lives, and thus reduce relevant economic burdens. Strategies such as psychological and behavioural intervention programs [40,41], policies such as stronger regulation of work hours and schedules, and national campaigns that promote public awareness of sleep health could all be effective [42,43]. Moreover, interventions should be tailored to the needs of the most affected groups, such as younger adult men, as identified in our study.

Our estimates need to be interpreted in light of several limitations. First, these estimates relied on self-reported sleep duration, which may overestimate objectively measured sleep duration [44] and is subject to recall bias and associated with individual health status [45]. The RRs used for estimating YLLs in this study were also based on self-reported measures. Future studies should use objective measures such as data collected by actigraphy or polysomnography to assess YLLs attributable to short sleep. Second, we assumed that the sleep duration category of individuals remained constant throughout the life course, which might not be the case for some individuals. Future research should track sleep duration trajectories longitudinally and investigate how the changes in sleep patterns are associated with mortality and YLLs [46]. Third, we relied on previous estimates of RRs from meta-analyses instead of calculating them based on individual-level data due to data availability and quality concerns. However, these estimates were unable to adjust for all potential confounding variables specific to our study population, such as socioeconomic status, lifestyle factors, and health status. Future studies should look for opportunities to obtain individual-level information to calculate the relative risks of death due to short sleep by sex and age groups or conduct updated meta-analyses for the specific population subgroups. Additionally, future studies should assess the impacts of other sleep indicators, such as sleep quality, on life expectancy.

Regarding economic cost estimations, the human capital approach had several assumptions. Primarily, it presumes that the economic value of living an additional year is solely based on an individual’s capacity to generate income through labour. It only calculated the monetary value of life years at working ages and did not consider non-labour contributions to the economy and society. Also, the estimation is sensitive to the productivity weights. These weights are unable to fully account for variations in productivity that are not directly related to age, such as those due to education or skill levels. A willingness-to-pay approach, for instance, would use the value of a statistical life year that is typically higher than the estimation of the human capital approach [47]. Furthermore, we only estimated costs associated with YLLs, but the economic cost can also involve direct health care costs and indirect work-related costs [5,48]. This remaining gap calls for a more comprehensive estimation of the overall economic cost attributable to insufficient sleep using various estimation approaches.

## CONCLUSIONS

Our results demonstrated that insufficient sleep was associated with a substantial number of YLLs in China. Furthermore, we provided quantified numbers of the economic cost of aggregate YLLs due to short sleep and showed that the economic cost increased over 3-fold from 2010 to 2018. This signified the importance of sleep health advocacy at the societal level.

## Additional material


Online Supplementary Document
