# Supplementary material for: Years of life lost due to insufficient sleep and associated economic burden in China from 2010–18
Source: J Glob Health. 2024 Apr 5;14:04076. doi: 10.7189/jogh.14.04076 (PMC10994670; doi:10.7189/jogh.14.04076)
Supplement: Online Supplementary Document [file jogh-14-04076-s001.pdf]

Table S1 Individual and aggregate years of life lost (YLL) by sex and age group in 2010, 2014, 2018

Year	Age group	Sex	YLL	Short sleep prevalence	Population size	Aggregate YLL
2010	20-24	Men	0·98	0·06	64,008,573	3,697,412·21
2010	25-29	Men	0·96	0·07	50,837,038	3,606,922·53
2010	30-34	Men	0·94	0·08	49,521,822	3,821,636·75
2010	35-39	Men	0·91	0·09	60,391,104	4,798,477·61
2010	40-44	Men	0·88	0·09	63,608,678	5,201,258·21
2010	45-49	Men	0·84	0·11	53,776,418	5,073,345·21
2010	50-54	Men	0·78	0·12	40,363,234	3,870,822·40
2010	55-59	Men	0·72	0·12	41,082,938	3,398,315·20
2010	60-64	Men	0·64	0·12	29,834,426	2,271,610·92
2010	65-69	Men	0·54	0·12	20,748,471	1,340,934·12
2010	70-74	Men	0·49	0·15	16,403,453	1,211,002·72
2010	75-79	Men	0·43	0·11	11,278,859	559,175·95
2010	80-84	Men	0·39	0·11	5,917,502	247,384·09
2010	85+	Men	0·41	0·12	2,857,250	145,649·28
2010	20-24	Women	0·78	0·05	63,403,945	2,234,174·36
2010	25-29	Women	0·76	0·04	50,176,814	1,556,435·13
2010	30-34	Women	0·75	0·05	47,616,381	1,721,681·75
2010	35-39	Women	0·74	0·06	57,634,855	2,531,941·06
2010	40-44	Women	0·72	0·08	61,145,286	3,574,409·49
2010	45-49	Women	0·70	0·10	51,818,135	3,583,003·06
2010	50-54	Women	0·67	0·12	38,389,937	3,136,985·36
2010	55-59	Women	0·63	0·11	40,229,536	2,808,290·71
2010	60-64	Women	0·58	0·14	28,832,856	2,282,077·60
2010	65-69	Women	0·51	0·14	20,364,811	1,457,939·58
2010	70-74	Women	0·47	0·15	16,568,944	1,212,562·90
2010	75-79	Women	0·43	0·15	12,573,274	783,455·23
2010	80-84	Women	0·39	0·13	7,455,696	368,587·47
2010	85+	Women	0·38	0·14	4,758,898	255,970·13
						Total = 66,751,461·03
2014	20-24	Men	0·95	0·05	57,045,012	2,568,831·83
2014	25-29	Men	0·94	0·06	60,585,158	3,645,992·77
2014	30-34	Men	0·91	0·07	50,823,601	3,024,463·46
2014	35-39	Men	0·89	0·09	50,804,136	4,213,138·34
2014	40-44	Men	0·86	0·11	63,364,964	6,028,100·73
2014	45-49	Men	0·82	0·12	61,380,779	6,245,023·99
2014	50-54	Men	0·77	0·15	48,017,032	5,436,160·53
2014	55-59	Men	0·71	0·19	41,096,107	5,577,414·53
2014	60-64	Men	0·64	0·21	37,446,472	4,955,592·34
2014	65-69	Men	0·54	0·22	25,027,981	2,919,433·82
2014	70-74	Men	0·49	0·23	17,673,966	1,984,802·61
2014	75-79	Men	0·44	0·21	12,383,212	1,139,807·23
2014	80-84	Men	0·39	0·24	7,283,455	694,005·52
2014	85+	Men	0·41	0·25	3,543,796	365,258·69
2014	20-24	Women	0·74	0·05	53,399,027	2,068,844·05

2014	25-29	Women	0·73	0·06	59,664,234	2,659,465·02
2014	30-34	Women	0·72	0·07	49,596,107	2,543,167·60
2014	35-39	Women	0·71	0·08	48,700,730	2,856,762·19
2014	40-44	Women	0·69	0·10	60,672,749	4,370,867·29
2014	45-49	Women	0·67	0·13	59,361,314	5,362,502·04
2014	50-54	Women	0·65	0·19	46,762,774	5,848,028·55
2014	55-59	Women	0·62	0·25	39,693,431	6,051,022·19
2014	60-64	Women	0·57	0·25	37,501,217	5,268,924·33
2014	65-69	Women	0·51	0·28	25,714,112	3,639,193·83
2014	70-74	Women	0·47	0·27	17,768,856	2,270,650·14
2014	75-79	Women	0·43	0·35	13,565,693	2,030,939·14
2014	80-84	Women	0·39	0·26	8,883,212	903,286·03
2014	85+	Women	0·40	0·27	5,810,219	621,385·29
						Total = 95,293,064·07
2018	20-24	Men	0·94	0·06	42,120,513	2,428,699·39
2018	25-29	Men	0·92	0·06	54,084,615	3,062,358·95
2018	30-34	Men	0·90	0·10	60,621,795	5,424,976·34
2018	35-39	Men	0·87	0·10	50,328,205	4,280,659·34
2018	40-44	Men	0·84	0·11	50,300,000	4,670,979·15
2018	45-49	Men	0·80	0·13	62,374,359	6,427,611·62
2018	50-54	Men	0·76	0·18	60,314,103	8,288,400·71
2018	55-59	Men	0·70	0·18	47,670,513	6,146,653·41
2018	60-64	Men	0·63	0·24	39,061,539	5,831,309·37
2018	65-69	Men	0·53	0·24	34,573,077	4,395,366·48
2018	70-74	Men	0·49	0·25	22,167,949	2,703,789·43
2018	75-79	Men	0·44	0·28	13,548,718	1,654,310·21
2018	80-84	Men	0·39	0·27	8,002,564	854,777·80
2018	85+	Men	0·41	0·24	4,797,436	464,024·00
2018	20-24	Women	0·73	0·04	36,750,000	1,118,235·06
2018	25-29	Women	0·72	0·06	50,711,539	2,022,899·56
2018	30-34	Women	0·71	0·09	59,853,846	3,745,223·79
2018	35-39	Women	0·70	0·11	49,291,026	3,660,648·93
2018	40-44	Women	0·69	0·13	48,474,359	4,406,056·36
2018	45-49	Women	0·67	0·17	60,216,667	6,697,165·74
2018	50-54	Women	0·64	0·22	59,075,641	8,260,537·65
2018	55-59	Women	0·61	0·25	47,289,744	7,306,767·17
2018	60-64	Women	0·56	0·30	38,774,359	6,512,567·29
2018	65-69	Women	0·51	0·29	36,050,000	5,382,864·96
2018	70-74	Women	0·47	0·34	23,556,410	3,784,348·72
2018	75-79	Women	0·43	0·39	15,438,462	2,571,562·09
2018	80-84	Women	0·40	0·36	10,360,256	1,492,972·43
2018	85+	Women	0·41	0·47	7,510,256	1,451,402·71
						Total = 115,047,168·66
